# Landscape Genetics Reveals Geographic Structuring of Locally Adapted Goat Populations from Brazil, Spain, and Ecuador

**DOI:** 10.3390/genes17050566

**Published:** 2026-05-15

**Authors:** Luis Antonio Castillo Cevallos, Laura Leandro da Rocha, Edgar Lenin Aguirre Riofrio, Amparo Martinez Martinez, Juan Vicente Delgado Bermejo, Maria Norma Ribeiro

**Affiliations:** 1 Facultad de Medicina Veterinaria y Agronomía, Universidad UTE, Km 4½ de la Vía Chone y Av. Italia, Santo Domingo 230102, Ecuador; 2 Programa de Pós-Graduação em Zootecnia, Universidade Federal Rural de Pernambuco, Av. Dom Manoel de Medeiros, s/n, Dois Irmãos, Recife 52171-900, Brazil; 3 Departamento de Zootecnia, Universidade Federal Rural de Pernambuco, Av. Dom Manoel de Medeiros, s/n, Dois Irmãos, Recife 52171-900, Brazil; laura.rocha@ufrpe.br (L.L.d.R.); maria.nribeiro@ufrpe.br (M.N.R.); 4 Facultad Agropecuaria y de Recursos Naturales Renovables, Carrera de Medicina Veterinaria, Universidad Nacional de Loja, Ciudad Universitaria Guillermo Falconí, Loja 110110, Ecuador; edgar.aguirre@unl.edu.ec; 5 Departamento de Genética, Universidad de Córdoba, Campus Universitario de Rabanales, 14014 Córdoba, Spain; ib2mamaa@uco.es (A.M.M.); id1debej@uco.es (J.V.D.B.)

**Keywords:** Creole goat, genetic conservation, genetic differentiation, microsatellite markers, population structure

## Abstract

**Background:** Locally adapted goat populations represent important reservoirs of genetic diversity and play a crucial role in the sustainability of livestock production systems, particularly in marginal environments. However, many of these populations are currently threatened by genetic erosion caused by crossbreeding with highly specialized commercial breeds. Although previous studies have described the genetic diversity of several goat populations from South America and the Iberian Peninsula, the influence of geographic factors on the genetic structure of these populations remains insufficiently understood. In this study, we investigated the influence of geographic distance and spatial factors on the genetic diversity, population structure, and relationships among locally adapted goat populations from Brazil, Spain, and Ecuador. **Methods:** A total of 561 goats representing 15 populations were genotyped using a panel of 23 microsatellite markers. The dataset included six locally adapted Brazilian breeds, three Spanish breeds, one Ecuadorian population (Chusca Lojana), four exotic breeds, and one undefined genotype group. Genetic diversity parameters, population structure, genetic relationships, and spatial genetic patterns were evaluated through a combination of population genetic and spatial analyses. **Results:** The locally adapted populations showed considerable levels of genetic diversity, with the Spanish (*H*_o_ = 0.629; *H*_e_ = 0.685) and Ecuadorian (*H*_o_ = 0.628; *H*_e_ = 0.704) populations displaying higher diversity than the Brazilian populations (*H*_o_ = 0.583; *H*_e_ = 0.628). Significant genetic differentiation was observed among geographic groups. A strong and significant correlation between genetic and geographic distances was detected when all local populations were considered (r = 0.77; R^2^ = 0.59; *p* < 0.001), as well as when only Brazilian populations were analyzed (r = 0.65; R^2^ = 0.43; *p* = 0.0075). Spatial analyses further identified potential genetic barriers that may restrict gene flow among certain populations. **Conclusions:** These findings suggest that geographic isolation plays an important role in shaping the genetic structure of locally adapted goat populations, while historical connections among Iberian and South American populations may also contribute to the observed genetic relationships. The integration of genetic and spatial information provides valuable insights for understanding the evolutionary dynamics of these populations and supports the development of more effective strategies for the conservation and sustainable management of goat genetic resources.

## 1. Introduction

The emergence of agriculture during the Neolithic transition, including the domestication of livestock, marked a pivotal point in human evolution. Among the first domesticated ungulates was the goat (*Capra hircus*), domesticated approximately 10,000 years ago in Southwest Asia from the wild bezoar (*Capra aegagrus*) [1,2]. Since then, goats have spread across the globe through human migrations and trade routes, undergoing adaptation to a wide range of agroclimatic conditions and production systems. Over time, processes such as founder effects, genetic drift, and local selection have contributed to the differentiation of numerous breeds and locally adapted populations observed today [3,4].

Locally adapted goat breeds play an essential role in the livelihoods of millions of people, particularly in marginal environments where intensive livestock production is not feasible. Their ability to tolerate heat stress, limited water availability, and poor-quality forage allows them to thrive in harsh environments and rugged landscapes [5,6]. In addition to their productive importance, these populations provide significant ecological and sociocultural benefits, including contributions to rural development, landscape management, and food security [7]. However, many locally adapted breeds are currently threatened by genetic erosion caused by indiscriminate crossbreeding with highly specialized commercial breeds or by replacement with more productive genotypes [8,9]. As a result, these populations represent valuable reservoirs of genetic diversity whose conservation and sustainable use are increasingly recognized as global priorities.

Genetic diversity studies provide essential information for understanding the origin, evolution, and management of animal genetic resources. Analyses of population structure, gene flow, and levels of genetic variability can support the design of effective strategies for conservation and genetic improvement [10]. Over the past decades, advances in molecular technologies have significantly enhanced the ability to characterize genetic variation within and between populations. Although single nucleotide polymorphism (SNP) technologies have become increasingly common, microsatellite markers remain widely used in studies of livestock genetic resources due to their high polymorphism, cost-effectiveness, and compatibility with historical datasets [11,12,13,14]. These markers have been successfully applied in numerous studies to investigate genetic diversity, population structure, and relationships among goat breeds in different regions of the world [15,16,17,18,19,20,21,22,23].

Genetic structuring and gene flow among populations are strongly influenced by spatial and environmental factors. Geographic distance, landscape heterogeneity, and natural barriers may restrict dispersal and connectivity among populations, shaping patterns of genetic differentiation over time [24]. In this context, the integration of genetic data with spatial information has given rise to the field of landscape genetics, which aims to understand how geographic and environmental features influence population structure and gene flow [25,26]. Geographic Information Systems (GIS) provide powerful tools for integrating spatial and genetic data, enabling researchers to evaluate how landscape features and geographic distance influence genetic connectivity among populations [27].

Although landscape genetics approaches are widely applied in studies of wild animal species, their use in livestock populations remains relatively limited [28,29,30,31]. In domestic species, most studies have focused primarily on assessing levels of genetic diversity and population structure, often without explicitly considering spatial factors that may influence gene flow and differentiation [32,33,34,35]. Consequently, the integration of genetic and spatial data in the study of livestock populations represents an important opportunity to improve our understanding of the evolutionary processes shaping genetic diversity in these species.

In the Americas, many locally adapted goat populations are believed to have originated from animals introduced during the colonial expansion of Iberian powers. Historical records indicate that goats were transported from the Iberian Peninsula to different regions of South America beginning in the 15th and 16th centuries, where they subsequently adapted to local environmental conditions and production systems. Over time, geographic isolation, genetic drift, and adaptation to diverse environments likely contributed to the differentiation of distinct Creole goat populations across the continent.

Several studies have previously evaluated the genetic diversity and population structure of locally adapted goat populations from Brazil, Ecuador, and Spain, including breeds such as Serrana Azul, Moxotó, Marota, Canindé, Repartida, Graúna, Murciana, Murciana-Granadina, Granadina, and the Ecuadorian Chusca Lojana population [8,15,22,36]. However, the extent to which geographic separation contributes to the observed genetic differentiation among these populations remains insufficiently explored. Understanding whether current patterns of genetic structure reflect primarily geographic isolation, historical dispersal processes, or a combination of both is essential for improving conservation strategies and management of these genetic resources.

Therefore, we hypothesized that geographic separation plays an important role in shaping the genetic structure of locally adapted goat populations, while historical connections among Iberian and South American goats may also contribute to the genetic relationships observed among breeds. Thus, the aim of this study was to investigate the influence of geographic factors on genetic diversity levels, population structure patterns, and genetic relationships among locally adapted goat populations from Brazil, Spain, and Ecuador through the integration of microsatellite genetic data and spatial information.

## 2. Materials and Methods

### 2.1. Goat Sampling

The dataset used [15,36] included 561 unrelated animals from six locally adapted populations in Brazil (BR), specifically Serrana Azul (SAZUL, *n* = 40), Moxotó (MOX, *n* = 40), Marota (MARO, *n* = 40), Canindé (CANIN, *n* = 40), Repartida (REPAR, *n* = 40), and Graúna (GRAU, *n* = 39), along with an undefined genotype standard group (SRD, *n* = 40). The SRD population represents animals with no defined breed pattern, commonly found in Brazilian production systems. This group was included as a reference population representing admixed goats resulting from historical crossbreeding processes. Additionally, it comprised three local goat breeds from Spain (ES)—namely, Murciana (MUR, *n* = 35), Murciana-Granadina (MG, *n* = 20), and Granadina (GRAN, *n* = 35)—and a native population from Ecuador (EC), known as Chusca Lojana (LOJ, *n* = 50). Lastly, four exotic breeds (EX) were included as an external comparison group—Alpine (ALP, *n* = 40), Boer (BOER, *n* = 40), Anglo-Nubian (ANG, *n* = 26), and Saanen (SAAN, *n* = 36)—totaling 15 populations.

### 2.2. Microsatellite Marker Genotyping

A panel of 23 microsatellite markers was analyzed according to the recommendations of the FAO [37] and the International Society for Animal Genetics (ISAG) for studies on genetic diversity in goats. The analyses were performed using previously collected biological samples and genotypic databases from locally adapted goat populations from Brazil, Spain, and Ecuador reported in previous studies [15,36]. Genomic DNA had been previously extracted from biological samples following standardized protocols previously described [15,36].

The microsatellite markers included in this study were *BM1329*, *BM6506*, *BM8125*, *BM1818*, *CSRD247*, *HSC*, *MM12*, *OarFCB48*, *SRCRSP8*, *INRA63*, *MAF209*, *ILSTS011*, *SPS115*, *TGLA122*, *BM6526*, *CSRM60*, *CSSM66*, *McM527*, *OarFCB11*, *OarFCB304*, *MAF65*, *ETH225*, and *ETH10*. Microsatellite amplification and multiplex organization followed previously standardized protocols described in the original source studies [15,36]. Fragment separation was conducted by capillary electrophoresis using ABI automatic sequencers (Applied Biosystems, Foster City, CA, USA), and allele calling was performed using the GeneScan (v3.1.2) and Genotyper (v2.5.2) software packages.

### 2.3. Statistical Data Analyses

The mean number of alleles (*N*_a_), observed heterozygosity (*H*_o_), and unbiased expected heterozygosity (*uH*_e_) were estimated using the GenAIEx software version 6.5 [38]. The number of loci deviating from Hardy–Weinberg equilibrium (*HWE*) proportions and the effective number of alleles (*N*_e_) were obtained using the Genepop software version 4.7.5 [39] and Popgene version 1.32 [40], respectively. Some loci presented occasional deviations from Hardy–Weinberg equilibrium in specific populations; however, no marker showed consistent deviation across all analyzed populations. Therefore, all loci were retained in the analyses to preserve comparability among datasets and because the observed deviations were compatible with expected population structure effects in locally adapted goat populations. The Fstat software version 2.9.4 [41] was employed to estimate allelic richness (*R*_t_) and Wright’s F statistics, with 95% confidence intervals for *F*_IS_ estimated using Genetix version 4.05 [42]. Arlequin version 3.5.2.2 [43] was utilized to obtain and graphically represent *F*_ST_ estimates between pairs of breeds.

A factorial correspondence analysis was conducted using Genetix version 4.05 [42]. Pairwise Nei’s genetic distances (*D*_A_) between populations [44] were estimated using Populations version 1.2.32 [45]. A neighbor-net network illustrating the relationships among breeds was constructed using Nei’s *D*_A_ distances and visualized with SplitsTree4 version 4.19.1 [46].

A Bayesian clustering approach implemented in Structure version 2.3.4 [47] was employed to examine the genetic structure and admixture levels of the 15 goat populations in this study. The most likely number of ancestral populations (*K*) was estimated using a burn-in period of 150,000, followed by 450,000 iterations of the Markov Chain Monte Carlo (MCMC). For each *K* value, ranging from 2 to 15, five independent runs were conducted. The alpha parameter, representing the degree of admixture, was inferred from the data using default settings and an admixture model with correlated allele frequencies [48]. The optimal *K* value was determined using the web-based Structure Selector tool [49] (available at: https://lmme.ac.cn/StructureSelector/, accessed on 12 March 2024) following the method of Evanno [50]. The resulting consensus Q matrix was obtained from five replicates of the optimal *K* and visualized with the web version of the POPHELPER package version 1.0.10 [51]. Major and minor clustering modes for each *K* value were verified using Clumpak [52].

Since the local populations (SAZUL, MOX, MARO, CANIN, REPAR, GRAU, MUR, MG, GRAN, and LOJ) represent the primary focus of analysis, genetic data were aggregated and linked to the centroid of each population’s distribution to assign each breed a unique location on the geographic map. These centroids were subsequently used in spatial model analyses [53]. Geographic coordinates of the populations were approximated using the Google Earth Pro geodatabase, version 7.3.6, which is particularly useful for providing coordinates in digital format.

A spatial analysis of the genetic structure of the ten locally adapted populations was performed using Bayesian analysis with the software Bayesian Analysis of Population Structure (BAPS), version 6.0 [54]. This analysis assumed that markers were unlinked and in Hardy–Weinberg equilibrium (*HWE*), followed by additional admixture analyses. The analysis incorporated the geographic coordinates of each population. The optimal number of ancestral populations (*K*) was determined after performing 20 replicates, with *K* values ranging from 2 to 10.

Network analysis was applied to examine connectivity patterns among the local populations, based on *F*_ST_ values [55] and without the assumption of predefined genetic groups, using the software EDENetworks, version 2.18 [56]. Populations were represented by nodes in the network, each differentiated by color, and the connections (links) between nodes were limited by a maximum distance automatically defined by the software. Links exceeding this threshold were removed. Connectivity degree, indicating the number of edges connecting a node, was used as a measure of how interconnected each population was with others. Additionally, betweenness centrality (BC), representing the number of shortest paths passing through a node among others, was calculated. To assess the robustness of BC values for the resolved network, the results were subjected to 10,000 bootstraps. The BC value distributions for nodes with the highest total BC were computed, taking into account all generated networks.

To assess isolation by distance and examine the correlation between genetic and geographic distances among pairs of local populations, a Mantel test with 10,000 iterations was conducted using the software Arlequin version 3.5.2.2 [43]. Following this, the computational geometric approach using Monmonier’s maximum difference algorithm was applied with the program Barrier, version 2.2 [57], to identify genetic discontinuities (barriers) between local populations. The matrix of Slatkin’s linearized genetic distances [*F*_ST_/(1 − *F*_ST_)], generated using the software Arlequin v. 3.5.2.2 [43], was used along with the geographic coordinates for each population. Additional spatial analyses were performed to detect more subtle differentiation patterns, focusing exclusively on local Brazilian breeds. Maps for spatial overlay of the genetic results were created using the geospatial data processing and graphics packages in R, version 4.3.1 [58].

## 3. Results

### 3.1. Genetic Diversity Within Populations

The estimates of genetic diversity for the populations considered in the study are summarized in Table 1. The local populations with the highest values for mean number of alleles, effective number of alleles, and allelic richness are the Ecuadorian LOJ and the Spanish GRAN, followed closely by the exotic ALP, which has similar values. The lowest values were observed in the BOER, MARO, and SAZUL breeds.

The exotic breeds ANG and ALP exhibit the highest levels of observed heterozygosity (*H*_o_) at 0.693 and expected heterozygosity (*H*_e_) at 0.724, respectively. However, among the local populations, the Spanish GRAN and MG and the Ecuadorian LOJ show the most significant levels of genetic diversity. Conversely, the least significant levels were found in the Brazilian MOX and SAZUL populations. All populations displayed loci with substantial deviations from Hardy–Weinberg equilibrium, ranging from one (ANG and GRAN) to eight (MG). An excess of homozygotes (*F*_IS_ > 0.10) was detected in some populations (SRD, MOX, MUR, and LOJ).

### 3.2. Genetic Relationships Between Populations

**Figure 1:**
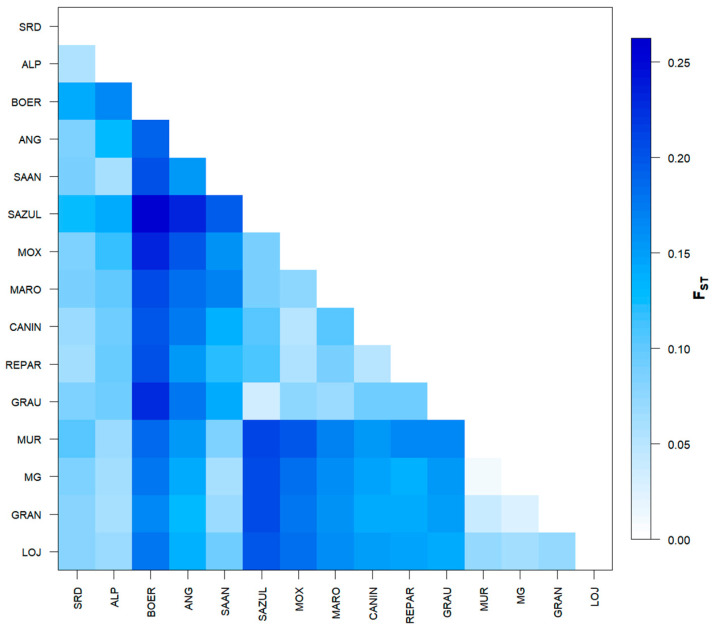
Graphical representation of the *F*_ST_ distance matrix by pairs among the 15 studied goat populations. The color values indicating the genetic distances are determined by the scale on the right side of the image.

All pairwise *F*_ST_ distance values (Figure 1 and Table S1) were significant at *p* < 0.05. The analysis revealed a substantial level of genetic differentiation between the BR breeds and those from ES, EC, and EX. In contrast, a moderate genetic differentiation was observed between the LOJ population from Ecuador and the Spanish populations, as well as between the SRD population and most of the other populations. When focusing on the local populations from Brazil, low genetic differentiation was confirmed between GRAU and SAZUL (*F*_ST_ = 0.03351), CANIN and REPAR (*F*_ST_ = 0.0496), MOX and CANIN (*F*_ST_ = 0.050), and between MOX and REPAR (*F*_ST_ = 0.0533). Conversely, the breeds from Spain exhibited low genetic differentiation among themselves.

**Figure 2:**
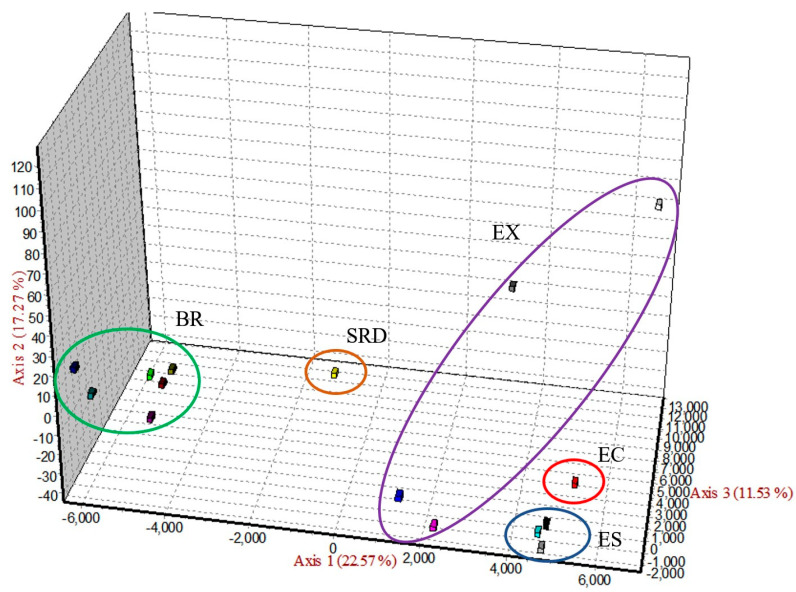
Factorial correspondence analysis of the 15 studied goat populations.

Through clustering methods, factorial correspondence analysis was implemented to assess the genetic structure of the populations included in the study. The results (Figure 2) showed that the BR populations are different from the ES populations, which, in turn, are more closely related to the native Ecuadorian population. On the other hand, among the EX breeds, ALP and SAAN appear to be more closely related to the ES breeds.

**Figure 3:**
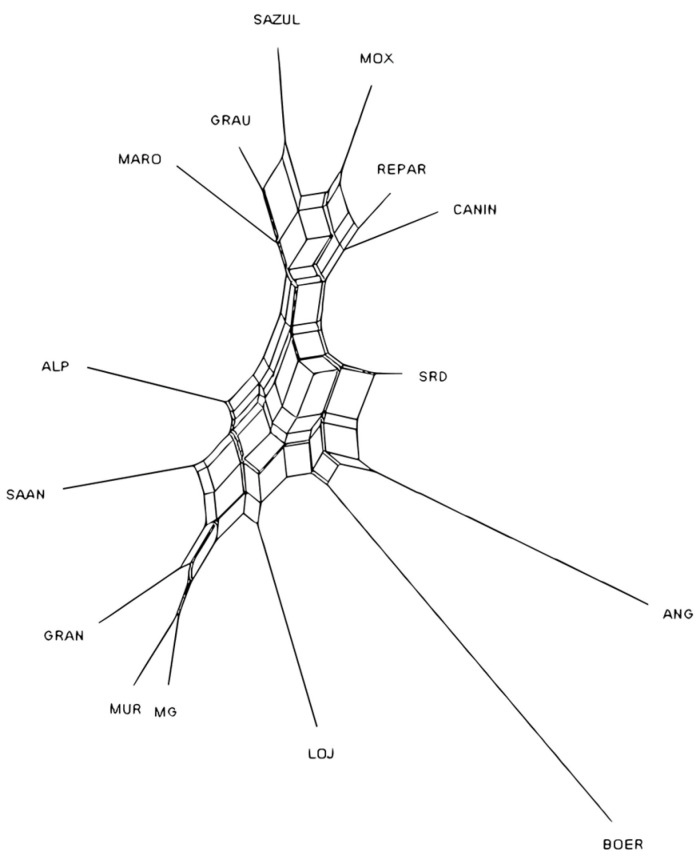
Neighbor-Net graph of Nei’s genetic distances *D*_A_ representing the racial relationships among the 15 goat populations.

The Neighbor-Net graph (Figure 3) constructed from the genetic distances *D*_A_ (Table S2) indicates the formation of different groups, where the BR populations form a narrow network distinct from the other geographic groups, which are located at the opposite end. The ES breeds also formed another closed racial group, with the LOJ population being the closest to them. The transboundary breed BOER was the most separated from the other racial groups. Furthermore, within the BR populations group, subgroups were identified, with MOX, REPAR, and CANIN on one side, and GRAU and SAZUL on the other.

### 3.3. Bayesian Clustering Analysis

**Figure 4:**
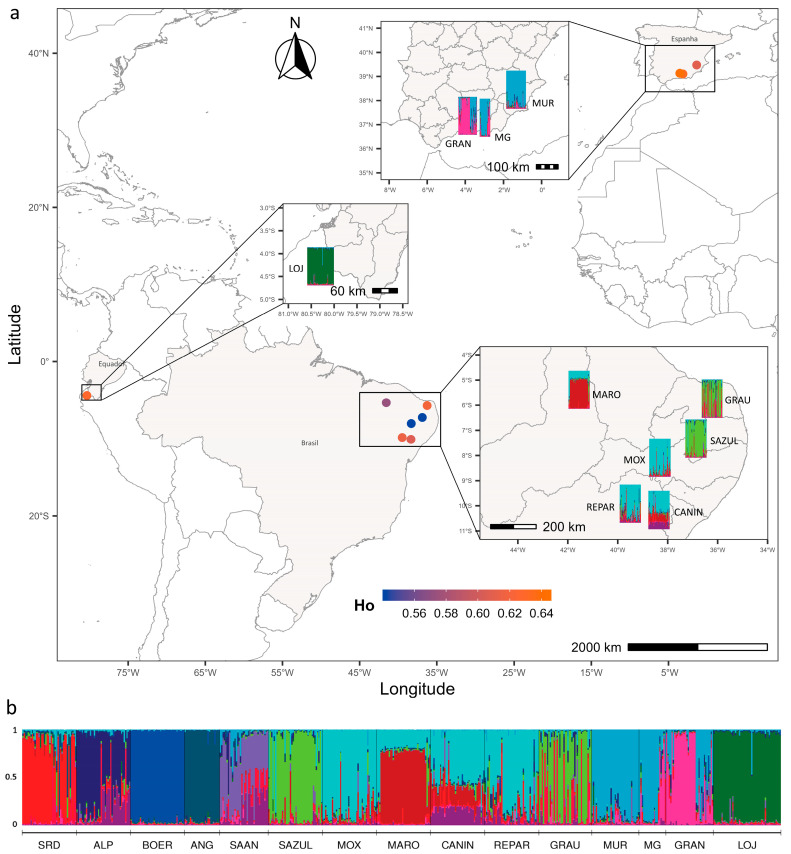
(**a**) Spatial overlap of the ten locally adapted goat populations with observed heterozygosity (circles) and Bayesian clustering (rectangles) inferred by the Structure program, based on genetic data from 561 individuals across the 15 populations for the optimal *K* = 13. Color gradients in the map represent observed heterozygosity (*H*_o_) values. (**b**) Bar plot of Bayesian clustering showing all populations included in the study, where each horizontal bar represents an individual, and the proportion of each color in the bar corresponds to the estimated membership coefficient for each individual, considering *K* = 13.

Based on the results obtained using the Bayesian approach implemented in Structure and the application of the Evanno method, it was inferred that *K* = 13 (Delta *K* = 19.8; see Figure S1) is the most likely number of ancestral populations contributing to the genetic variability observed in the 15 populations studied. The proportional values of the inferred ancestral population contributions for each breed are presented in Table S3. The graphical representation of the assumed ancestral population contributions for each individual (q) of the 15 studied breeds, considering *K* = 13, is shown in Figure 4. Among the locally adapted populations, the LOJ from EC clearly differentiated, showing the highest contribution from a single ancestral population. In turn, the Brazilian populations, SAZUL and GRAU shared a significant proportion of the same ancestral group. Similarly, MOX, REPAR, and CANIN shared a substantial contribution from one ancestral group, though it was less pronounced in the latter. The MARO breed appeared somewhat more isolated from the other populations, with a primary contribution from a different genetic group.

Significant spatial patterns in genetic structure were observed among the locally adapted populations (Figure 4a). As expected, the population groups from BR, ES, and EC exhibit genetic structuring consistent with their respective geographic regions. Focusing on the BR populations, it was observed that groups sharing higher proportions of ancestral genetic contributions also follow a defined spatial structuring, being geographically closer to each other. In this context, a strong correlation was evident between genetic (*F*_ST_) and geographic distances between pairs of breeds (r = 0.77; R^2^ = 0.59; *p* < 0.001), when considering the local breeds from BR, ES, and EC. When considering only the BR populations, the correlation was lower (r = 0.65); however, geographic distances still explained a significant proportion of the genetic variability among the populations (R^2^ = 0.43; *p* = 0.007500).

**Figure 5:**
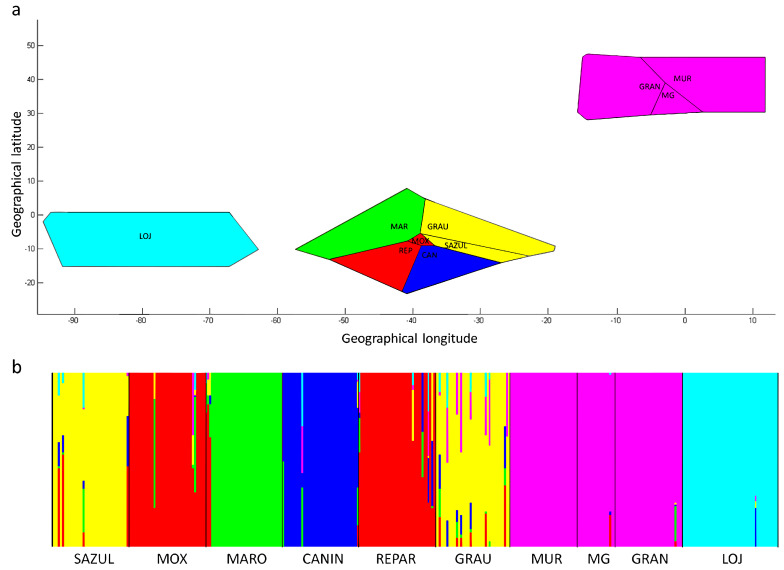
Results of the genetic structure analysis considering the local populations from BR, ES, and EC, using the BAPS program. (**a**) Results of the Bayesian population structure analysis with the spatial model applied. (**b**) Cluster admixture analysis of the identified clusters (*K* = 6).

Based on the Bayesian population structure analysis with spatial modeling implemented in the BAPS program, considering the BR, ES, and EC populations, the optimal partition of the data (*p* = 0.98264) into six genetic groups was established, represented by differently colored Voronoi polygons (Figure 5a). This structure yielded the highest maximum likelihood value among the partitions (−26,891.442). Spatial clustering was observed, with limited admixture among the genetic groups (Figure 5a,b), where all ES breeds were grouped into the same cluster. Among the BR populations, SAZUL and GRAU as well as MOX and REPAR were placed in the same genetic groups. These results were consistent with those obtained using the Structure program.

### 3.4. Network Analysis and Genetic Barriers

**Figure 6:**
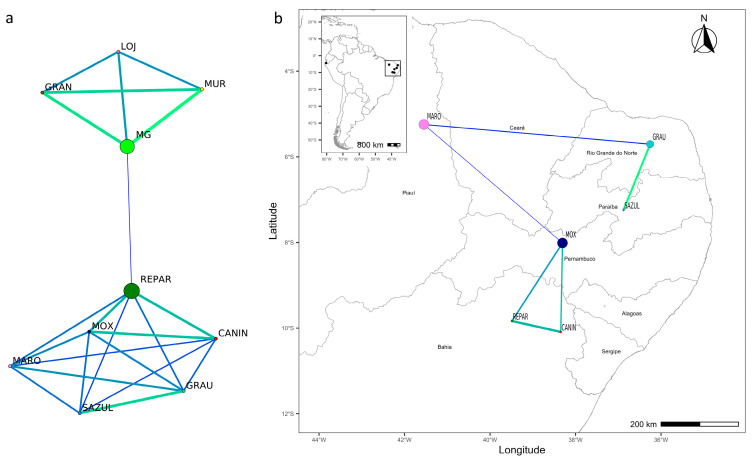
Genetic network (based on *F*_ST_ values) generated in the EDENetwork software considering: (**a**) the populations from BR, ES, and EC, and (**b**) the populations from BR. Edge thickness is inversely correlated with *F_ST_* values, with thicker edges representing less significant pairwise *F*_ST_ values. Node size is proportional to betweenness centrality values. Different colors represent distinct genetic connections identified by the EDENetwork analysis.

The network analysis supports the previously found results. The *F*_ST_ network revealed two main groups (Figure 6a), with the first containing the populations from ES and EC, and the second containing the populations from BR, with no disconnected individual components (i.e., no absence of edges connecting them to other sampling sites). When analyzing the group from BR independently (Figure 6b), more visible connectivity patterns were observed, with the highest betweenness centrality values for the MOX and MARO populations. Evidence of restricted gene flow was observed between MARO and other populations (SAZUL, CANIN, and REPAR). On the other hand, a higher degree of connectivity was found between the GRAU and SAZUL populations, suggesting a high level of gene flow between them. The MOX, CANIN, and REPAR populations also showed a significant level of connectivity. Additionally, high gene flow was observed among the ES breeds, especially between MUR and MG, a result expected due to the history of formation of these two breeds.

**Figure 7:**
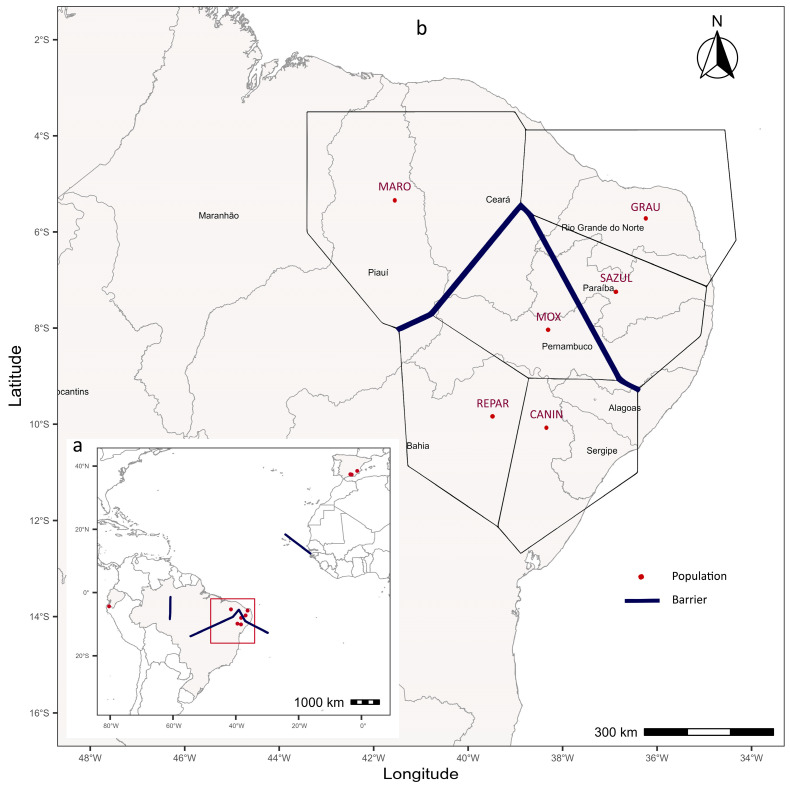
Most likely genetic barriers (discontinuities) identified using the Barrier software, (**a**) considering the local populations from BR, ES, and EC, and (**b**) only the populations from BR (main map).

Three genetic barriers were identified in our dataset (Figure 7a). The first was observed between the geographic groups from BR and EC; the second, between the groups from BR and ES; and the third, within the BR population group. Figure 7b provides a more detailed view of the barrier identified among the BR populations, indicating a potential genetic boundary that separates the MOX, REPAR, and CANIN group from the group formed by the SAZUL and GRAU populations, as well as showing a certain level of isolation for MARO. These barriers are consistent with the gene flow restrictions shown in the genetic network analysis (Figure 6).

## 4. Discussion

### 4.1. Genetic Diversity of Locally Adapted Goat Populations

The locally adapted goat populations analyzed in this study exhibited considerable levels of genetic diversity, reinforcing their importance as reservoirs of genetic variability. Allelic richness (*R_t_*), which is less influenced by sample size differences among populations, also revealed considerable levels of genetic diversity among the locally adapted goat populations, reinforcing their importance as reservoirs of adaptive genetic variation. Among the populations evaluated, the Spanish breeds and the Ecuadorian Chusca Lojana population presented the highest heterozygosity values, whereas some Brazilian populations such as Moxotó and Serrana Azul showed comparatively lower diversity levels. Similar levels of genetic variability have been reported in previous studies evaluating goat populations using microsatellite markers, which highlighted the presence of substantial genetic diversity within locally adapted breeds [19,21,22].

The relatively high genetic diversity observed in several populations may be associated with traditional management systems under which goats are commonly raised. In many regions, goats are maintained under extensive production systems with limited artificial selection and occasional animal exchange between herds, which may contribute to maintaining genetic variability within populations. Conversely, reduced diversity observed in some populations may be related to factors such as geographic isolation, reduced effective population sizes, or historical demographic events that limited gene flow.

From a conservation perspective, maintaining adequate levels of genetic diversity within locally adapted breeds is essential to ensure their long-term viability and adaptive potential. Genetic diversity constitutes a fundamental component of evolutionary resilience, enabling populations to respond to environmental changes, disease pressures, and evolving production conditions. Therefore, the genetic characterization of these populations represents an important step for guiding conservation and breeding strategies aimed at preserving animal genetic resources [8].

### 4.2. Genetic Relationships Among Goat Populations

The analyses of genetic differentiation and population relationships revealed clear structuring among the studied populations. Brazilian populations formed a relatively distinct cluster compared with Iberian breeds and the Ecuadorian population, suggesting the existence of geographic and historical factors influencing the differentiation among these groups. Interestingly, the Ecuadorian Chusca Lojana population showed closer genetic relationships with the Spanish breeds than with the Brazilian populations.

This pattern may reflect historical processes associated with the introduction of goats into South America during the colonial period. Goats were transported from the Iberian Peninsula to several regions of the Americas beginning in the 15th and 16th centuries, where they subsequently adapted to diverse environmental conditions and production systems. Over time, processes such as genetic drift, geographic isolation, and adaptation to local environments likely contributed to the differentiation of distinct Creole populations across the continent.

Previous genetic studies have reported similar relationships between Iberian and Latin American goat populations, suggesting a shared historical origin followed by regional diversification [8,22]. These findings support the hypothesis that many South American goat populations may retain genetic signatures of their Iberian ancestors while also reflecting the effects of local adaptation and population isolation.

The differentiation observed among Brazilian populations also reflects the complex demographic history of these breeds. Several Brazilian goat populations developed under distinct ecological conditions and production systems distributed across wide geographic areas. Consequently, geographic separation and limited connectivity between production regions may have promoted the emergence of differentiated genetic structures among populations.

### 4.3. Influence of Geographic Factors on Population Structure

The results of the Mantel test indicated a significant correlation between genetic and geographic distances among locally adapted populations, suggesting the presence of an isolation-by-distance pattern. This finding indicates that geographic separation may restrict gene flow among populations and contribute to the genetic differentiation observed among breeds.

Similar patterns have been reported in studies of livestock populations, where geographic distance and landscape characteristics influence population structure and genetic differentiation [33,34]. In traditional livestock production systems, animal exchange often occurs within relatively restricted geographic areas, meaning that large distances or natural barriers can effectively limit gene flow between populations.

Spatial analyses conducted in the present study also identified potential genetic barriers that may limit connectivity among certain populations. These barriers may arise from a combination of geographic, environmental, and management-related factors that influence animal movement. Landscape features such as mountain ranges, climatic gradients, or differences in production systems may therefore contribute to shaping patterns of genetic differentiation.

The integration of spatial and genetic analyses thus provides valuable insights into the processes influencing the distribution of genetic diversity across livestock populations. Although landscape genetics approaches are widely used in studies of wild species, their application in livestock populations remains relatively limited. Nevertheless, these approaches offer important opportunities to better understand the spatial dynamics of gene flow and population structure in domesticated animals [33].

### 4.4. Implications for Conservation of Goat Genetic Resources

The results obtained in this study highlight the importance of locally adapted goat populations as valuable reservoirs of genetic diversity. These populations represent important genetic resources for the sustainability of livestock production systems, particularly in regions where environmental conditions limit the use of highly specialized commercial breeds.

Locally adapted goats often possess adaptive traits that enable them to survive under challenging environmental conditions, including tolerance to heat stress, resistance to diseases and parasites, and the ability to utilize low-quality feed resources. Maintaining the genetic diversity present in these populations is therefore essential for preserving their adaptive potential and ensuring the long-term sustainability of goat production systems [8].

Understanding the spatial distribution of genetic diversity and the factors influencing gene flow among populations can contribute to the development of more effective conservation strategies. In this context, integrating genetic and spatial information provides an important framework for identifying priority populations for conservation and for designing management strategies aimed at maintaining connectivity among populations while preserving their unique genetic characteristics.

Although microsatellite markers remain informative for assessing genetic diversity, future studies using genome-wide SNP markers could provide higher resolution insights into the demographic history and adaptive variation of these populations. In addition, expanding sampling to other Creole goat populations across South America would further improve our understanding of the spatial dynamics of genetic diversity.

## 5. Conclusions

This study demonstrates that geographic factors play a significant role in shaping the genetic structure of locally adapted goat populations from Brazil, Spain, and Ecuador. The integration of genetic and spatial analyses revealed patterns of isolation by distance and potential barriers to gene flow among populations. These findings provide important insights into the evolutionary dynamics of these populations and contribute to the development of more effective conservation strategies for goat genetic resources.

## Figures and Tables

**Table 1:** Mean number of alleles (*N*_a_), effective number of alleles (*N*_e_), allelic richness (*R*_t_), observed heterozygosity (*H*_o_), unbiased expected heterozygosity (*uH*_e_), number of loci with deviations from Hardy–Weinberg proportions (*HWE*), and inbreeding coefficient (*F*_IS_) with their confidence intervals (*F*_IS_ *IC*) for the 15 goat populations included in the study.

Breeds	*N*	*N_a_*	*N_e_*	*R_t_*	*H_o_*	*uH_e_*	*HWE*	*F_IS_*	*F_IS_ IC (95%)*
SRD	40	6.957	4.078	6.188	0.636	0.710	7	0.106	(0.07656–0.12715)
ALP	40	7.304	4.429	6.380	0.661	0.724	6	0.088	(0.04826–0.09924)
BOER	40	5.087	2.870	4.515	0.594	0.624	4	0.05	(0.02082–0.06907)
ANG	26	5.217	2.947	4.807	0.693	0.636	1	−0.092	(−0.16167–−0.06605)
SAAN	36	7.174	3.811	6.138	0.630	0.666	6	0.055	(−0.00346–0.05501)
SAZUL	40	5.913	2.743	4.832	0.541	0.576	3	0.062	(0.02147–0.07877)
MOX	40	5.826	3.037	4.941	0.540	0.623	8	0.135	(0.09084–0.13648)
MARO	40	5.696	2.955	4.839	0.573	0.608	4	0.058	(0.01667–0.05831)
CANIN	40	5.957	3.274	5.205	0.607	0.655	4	0.074	(0.05001–0.10379)
REPAR	40	6.696	3.446	5.666	0.615	0.663	5	0.074	(0.04622–0.09162)
GRAU	39	6.739	3.209	5.589	0.620	0.644	3	0.039	(0.00696–0.05622)
MUR	35	6.826	3.724	5.896	0.609	0.677	4	0.102	(0.04144–0.10419)
MG	20	6.391	3.746	6.131	0.632	0.692	8	0.089	(0.00743–0.13477)
GRAN	35	7.304	3.986	6.391	0.646	0.686	1	0.06	(0.02566–0.07376)
LOJ	50	7.826	4.291	6.332	0.628	0.704	7	0.109	(0.07518–0.14594)

SRD, Undefined genotype standard group; ALP, Alpine; BOER, Boer; ANG, Anglo-Nubian; SAAN, Saanen; SAZUL, Serrana Azul; MOX, Moxotó; MARO, Marota; CANIN, Canindé; REPAR, Repartida; GRAU, Graúna; MUR, Murciana; MG, Murciana-Granadina; GRAN, Granadina; LOJ, Chusca Lojana. *HWE*, Hardy–Weinberg equilibrium for *p* < 0.05.

## Data Availability

Part of the raw data used in this study is protected by intellectual property rights, as it forms part of a private collection developed by one of the co-authors. Access to this data is regulated by internal policies that prevent public dissemination. These restrictions are in place to ensure compliance with institutional guidelines and to maintain the integrity and proper management of the dataset.

## Supplementary Materials

The following supporting information can be downloaded at: https://www.mdpi.com/article/10.3390/genes17050566/s1, Figure S1: Modal distribution of Delta *K* values according to the method of Evanno [50], estimated for *K* = 2 to *K* = 15 across the 15 goat populations included in the study; Figure S2: Structural clustering plots for the 15 goat populations, showing both minor and major modes for the data. Runs were conducted for *K* = 2 to 15 with 5 replicates, with the division of runs by mode provided below the plots. In the chart, each horizontal bar represents an individual, and the color proportions within each bar correspond to the proportion of the individual’s genotype assigned to a specific cluster; Figure S3: Distribution of betweenness centrality values for populations generated by bootstrapping using EDENetworks version 2.18; Table S1: Pairwise *F*_ST_ values among populations; Table S2: Pairwise population matrix of Nei genetic distances among populations; Table S3: Estimated membership coefficients from STRUCTURE (*K* = 13) for the genetic pools of the 15 goat populations.

## Abbreviations

The following abbreviations are used in this manuscript:

SRD	Undefined genotype/admixed population
BR	Brazil
ES	Spain
EC	Ecuador
EX	Exotic breeds
SAZUL	Serrana Azul
MOX	Moxotó
MARO	Marota
CANIN	Canindé
REPAR	Repartida
GRAU	Graúna
MUR	Murciana
MG	Murciana-Granadina
GRAN	Granadina
LOJ	Chusca Lojana
ALP	Alpine
BOER	Boer
ANG	Anglo-Nubian
SAAN	Saanen
*N*_a_	Mean number of alleles
*N*_e_	Effective number of alleles
*R*_t_	Allelic richness
*H*_o_	Observed heterozygosity
*H*_e_	Expected heterozygosity
*uH*_e_	Unbiased expected heterozygosity
*F*_IS_	Inbreeding coefficient
*F*_ST_	Fixation index (genetic differentiation)
*D*_A_	Nei’s genetic distance
*HWE*	Hardy–Weinberg equilibrium
MCMC	Markov Chain Monte Carlo
FCA	Factorial correspondence analysis
BAPS	Bayesian Analysis of Population Structure
BC	Betweenness centrality
SSR	Simple sequence repeats (microsatellites)
