# Supplementary material for: Landscape Genetics Reveals Geographic Structuring of Locally Adapted Goat Populations from Brazil, Spain, and Ecuador
Source: Genes (Basel). 2026 May 15;17(5):566. doi: 10.3390/genes17050566 (PMC13205470; doi:10.3390/genes17050566)
Supplement: Supplementary file 1 [file genes-17-00566-s001.zip › genes-4303837-supplementary.pdf]

Supplementary Materials

Landscape Genetics Reveals Geographic Structuring of Locally Adapted Goat Populations from Brazil, Spain, and Ecuador

Luis A. Castillo, Laura L. da Rocha, Edgar L. Aguirre, Amparo Martínez, Juan V. Delgado and Maria N. Ribeiro

Table S1. Pairwise F_{ST} values among populations.

Pop. Code	SRD	ALP	BOER	ANG	SAAN	SAZUL	MOX	MARO	CANIN	REPAR	GRAU	MUR	MG	GRAN	LOJ
SRD	0														
ALP	0.05735	0													
BOER	0.14171	0.16419	0												
ANG	0.08551	0.12926	0.19137	0											
SAAN	0.08906	0.05817	0.20193	0.15369	0										
SAZUL	0.12328	0.14033	0.2623	0.23188	0.19384	0									
MOX	0.08429	0.11885	0.23028	0.1996	0.15876	0.08663	0								
MARO	0.08697	0.1011	0.20592	0.18337	0.17109	0.08784	0.07485	0							
CANIN	0.06854	0.0928	0.19805	0.17373	0.13604	0.10563	0.05	0.10342	0						
REPAR	0.06526	0.09455	0.20448	0.15271	0.11898	0.10726	0.0533	0.08908	0.0496	0					
GRAU	0.08338	0.09194	0.22542	0.17966	0.14343	0.03351	0.07685	0.06839	0.09317	0.09028	0				
MUR	0.10411	0.06767	0.18776	0.1524	0.08403	0.21166	0.19866	0.1719	0.15519	0.16414	0.16538	0			
MG	0.08513	0.06282	0.17637	0.13982	0.0574	0.20702	0.18124	0.16349	0.14539	0.13691	0.15374	0.00945	0		
GRAN	0.08081	0.05968	0.1664	0.13	0.06965	0.20532	0.17892	0.15847	0.14342	0.14098	0.14983	0.03727	0.02521	0	
LOJ	0.08078	0.06732	0.17702	0.13723	0.09259	0.19917	0.18364	0.16378	0.14949	0.14673	0.14146	0.0728	0.06379	0.07354	0

$p < 0.05$ - ns. (not significant).

Table S2. Pairwise population matrix of Nei genetic distances among populations.

Pop. Code	SRD	ALP	BOER	ANG	SAAN	SAZUL	MOX	MARO	CANIN	REPAR	GRAU	MUR	MG	GRAN	LOJ
SRD	0														
ALP	0.181447	0													
BOER	0.288406	0.345812	0												
ANG	0.193282	0.307786	0.353365	0											
SAAN	0.195905	0.162823	0.349351	0.314537	0										
SAZUL	0.189562	0.230185	0.410214	0.370041	0.256381	0									
MOX	0.148759	0.234945	0.398166	0.336555	0.257692	0.128066	0								
MARO	0.149431	0.203047	0.366567	0.303762	0.256744	0.145308	0.112269	0							
CANIN	0.138742	0.205143	0.341693	0.315928	0.229448	0.145825	0.102428	0.146429	0						
REPAR	0.115944	0.201104	0.341425	0.262324	0.219673	0.143519	0.086642	0.121914	0.095627	0					
GRAU	0.158229	0.173165	0.38893	0.332123	0.190274	0.085956	0.132655	0.132192	0.148186	0.143118	0				
MUR	0.214897	0.196901	0.351718	0.30636	0.175253	0.310966	0.311124	0.279505	0.262024	0.26196	0.250978	0			
MG	0.203402	0.209027	0.323339	0.310174	0.164976	0.30081	0.310715	0.272638	0.26549	0.241653	0.243028	0.066457	0		
GRAN	0.205233	0.18699	0.311559	0.282991	0.150967	0.303316	0.306819	0.27515	0.255186	0.253246	0.237564	0.107653	0.102903	0	
LOJ	0.198517	0.209449	0.340168	0.29127	0.213123	0.31433	0.324414	0.295649	0.28571	0.269384	0.269046	0.182784	0.193884	0.204054	0

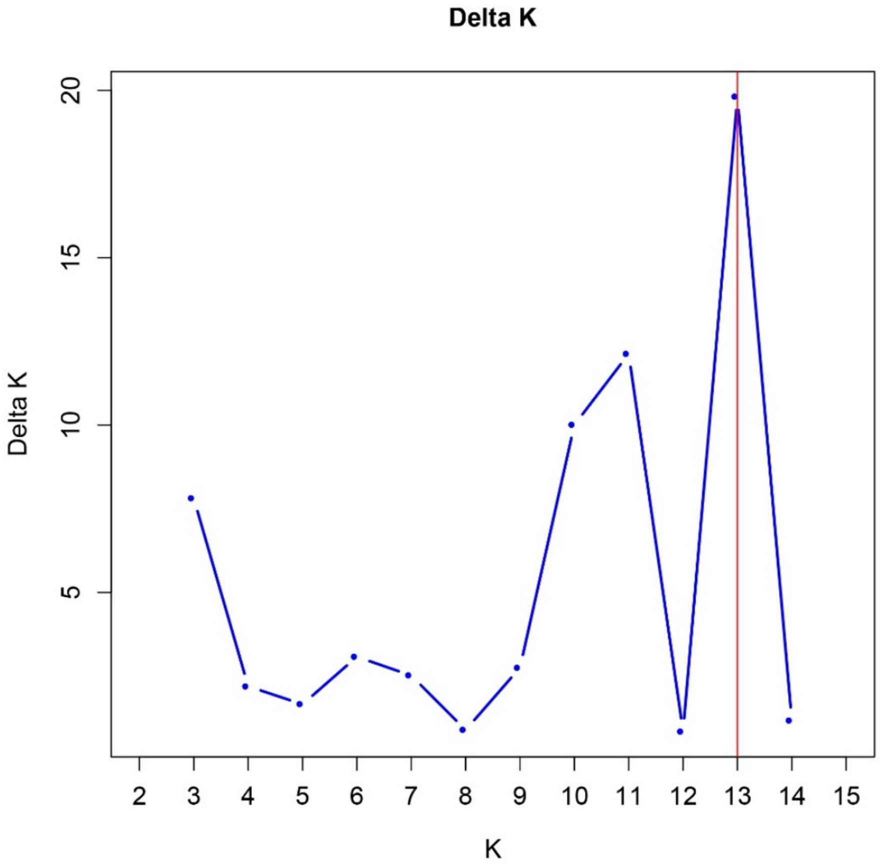

Figure S1. Modal distribution of Delta *K* values according to the method of Evanno [50], estimated for *K* = 2 to *K* = 15 across the 15 goat populations included in the study.

Table S3. Estimated membership coefficients from STRUCTURE (*K*=13) for the genetic pools of the 15 goat populations.

Pop. Code	Number of individuals	Cluster 1	Cluster 2	Cluster 3	Cluster 4	Cluster 5	Cluster 6	Cluster 7	Cluster 8	Cluster 9	Cluster 10	Cluster 11	Cluster 12	Cluster 13
SRD	40	0.0161	0.0371	0.0171	0.0646	0.0061	0.0154	0.0316	0.0107	0.0243	0.6668	0.0857	0.013	0.0115
ALP	40	0.0095	0.0163	0.0072	0.0057	0.6496	0.0109	0.0179	0.0165	0.0176	0.0233	0.0674	0.0106	0.1475
BOER	40	0.0049	0.0034	0.952	0.0055	0.0035	0.0037	0.003	0.0038	0.0037	0.0044	0.0041	0.0051	0.0029
ANG	26	0.0037	0.0048	0.0149	0.9255	0.007	0.0075	0.0032	0.0051	0.0053	0.0079	0.0044	0.0054	0.0054
SAAN	36	0.0645	0.0067	0.0071	0.012	0.0521	0.0069	0.007	0.5352	0.0069	0.0123	0.0923	0.0254	0.1715
SAZUL	40	0.0093	0.1068	0.0035	0.0058	0.0145	0.0116	0.7469	0.0169	0.0313	0.0118	0.0127	0.0079	0.0211
MOX	40	0.008	0.8027	0.0028	0.0072	0.0069	0.0055	0.0249	0.0151	0.0751	0.0123	0.0179	0.0048	0.0169
MARO	40	0.0052	0.2325	0.0043	0.0057	0.008	0.006	0.0365	0.0071	0.654	0.0179	0.0092	0.0061	0.0074
CANIN	40	0.0124	0.5054	0.0093	0.008	0.0091	0.0065	0.0182	0.0225	0.182	0.0375	0.0157	0.011	0.1623
REPAR	40	0.0133	0.6776	0.0111	0.0221	0.0145	0.013	0.0361	0.0155	0.0585	0.0573	0.0202	0.0123	0.0486
GRAU	39	0.0165	0.0254	0.0038	0.0075	0.0435	0.0068	0.5904	0.0264	0.0293	0.028	0.1732	0.0198	0.0294
MUR	35	0.8518	0.0075	0.0058	0.0066	0.0194	0.0146	0.0055	0.0249	0.0081	0.0124	0.015	0.0205	0.0079
MG	20	0.6804	0.0118	0.0144	0.0054	0.0163	0.0103	0.0077	0.0195	0.009	0.0295	0.0303	0.1517	0.0138
GRAN	35	0.2329	0.0067	0.0147	0.012	0.0258	0.0078	0.0061	0.0494	0.0095	0.0131	0.016	0.5956	0.0105
LOJ	50	0.0199	0.0051	0.0054	0.0063	0.0079	0.9004	0.0041	0.0139	0.0051	0.008	0.0076	0.0069	0.0093

Results from the STRUCTURE software

Major modes

K=2

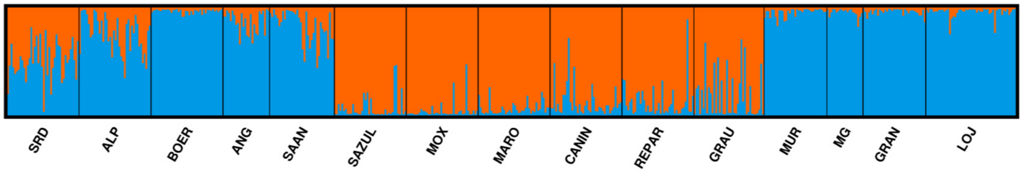

K=3

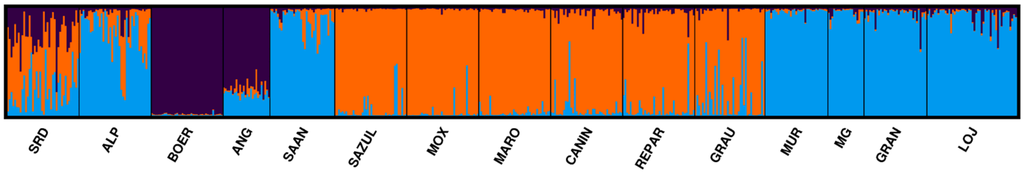

K=4

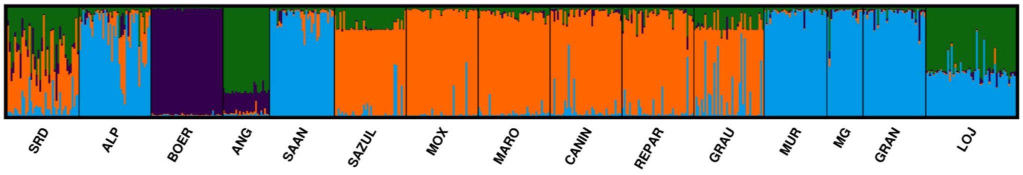

K=5

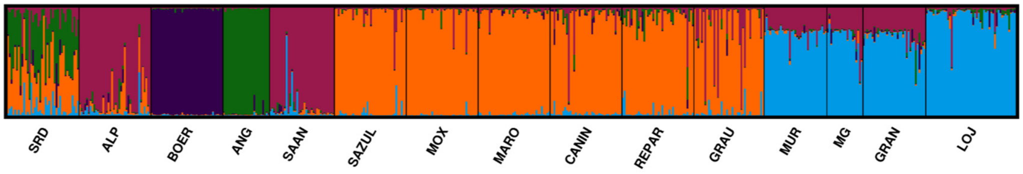

K=6

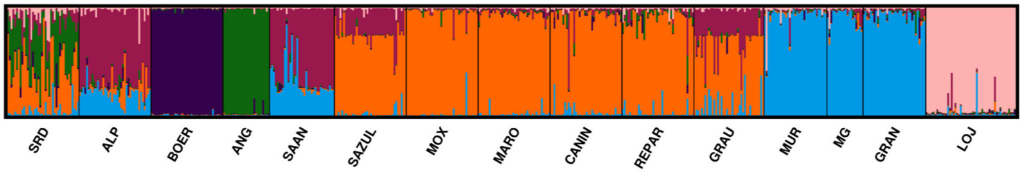

K=7

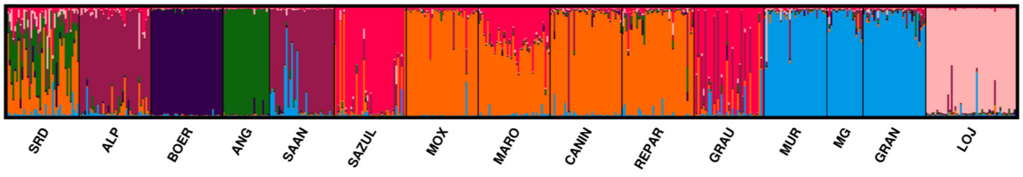

K=8

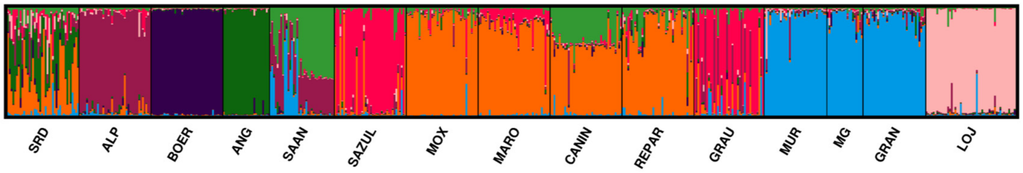

K=9

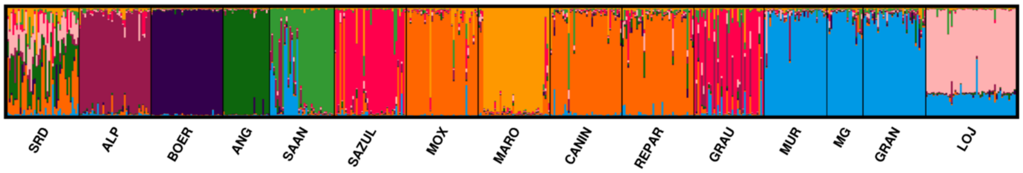

K=10

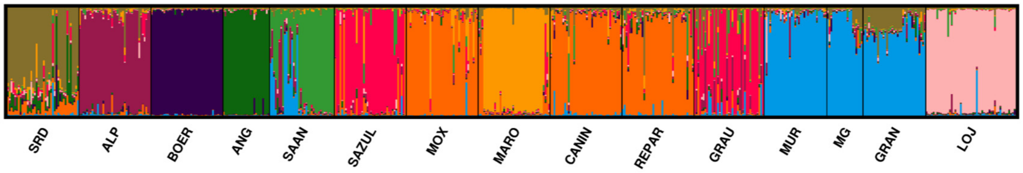

K=11

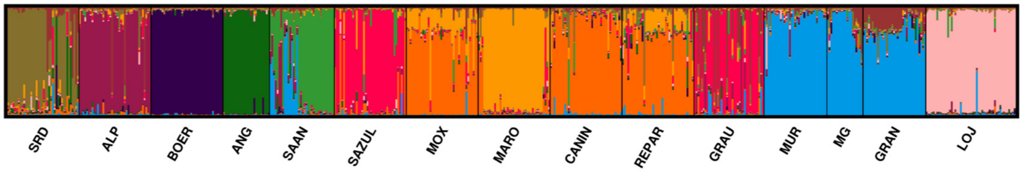

K=12

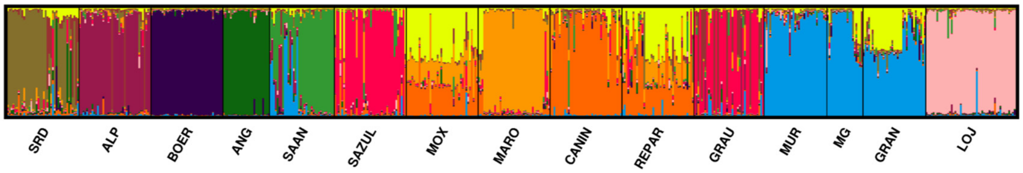

K=13

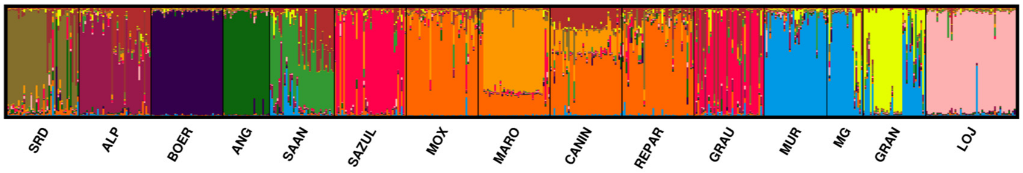

K=14

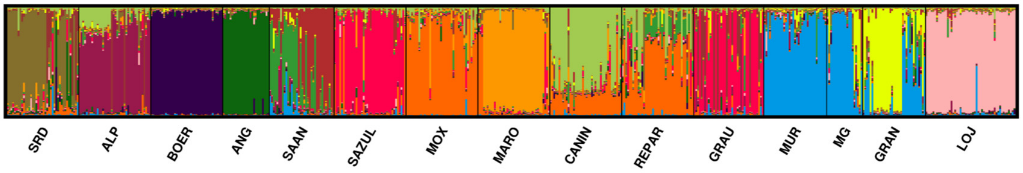

K=15

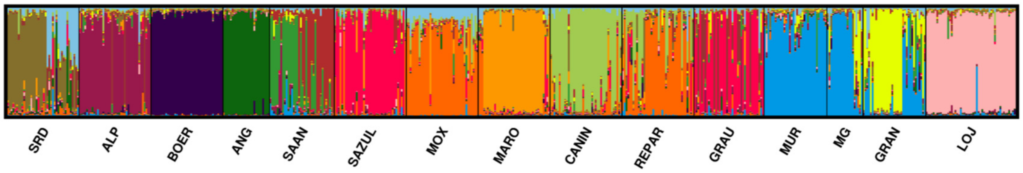

Minor modes

K=6

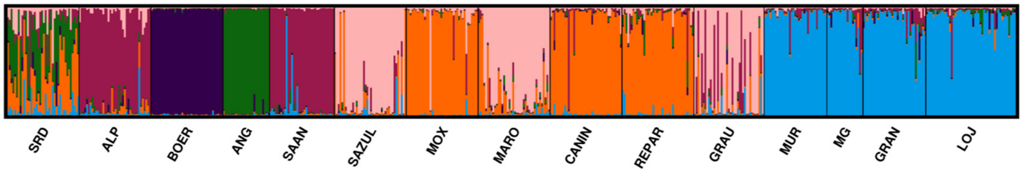

K=7

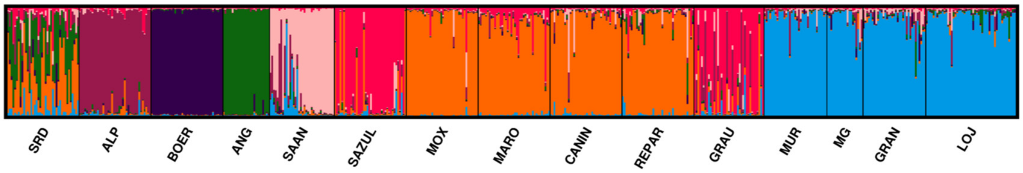

K=8

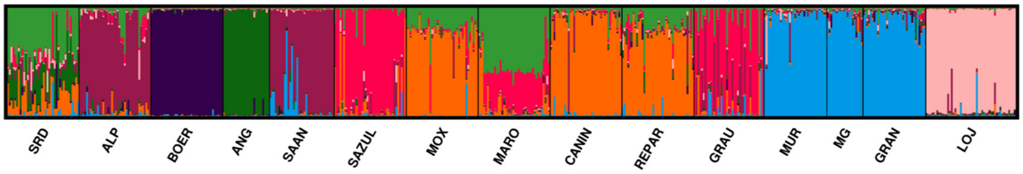

K=15

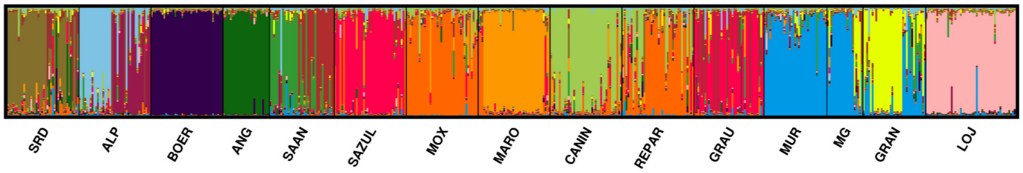

Division of runs by mode:

K=2 5/5
K=3 5/5
K=4 5/5
K=5 5/5
K=6 4/5, 1/5
K=7 3/5, 2/5
K=8 3/5, 2/5
K=9 5/5
K=10 5/5
K=11 5/5
K=12 5/5
K=13 5/5
K=14 5/5
K=15 3/5, 2/5

Figure S2. Structural clustering plots for the 15 goat populations, showing both minor and major modes for the data. Runs were conducted for K = 2 to 15 with 5 replicates, with the division of runs by mode provided below the plots. In the chart, each horizontal bar represents an individual, and the color proportions within each bar correspond to the proportion of the individual's genotype assigned to a specific cluster.

Results from the EDENetworks software

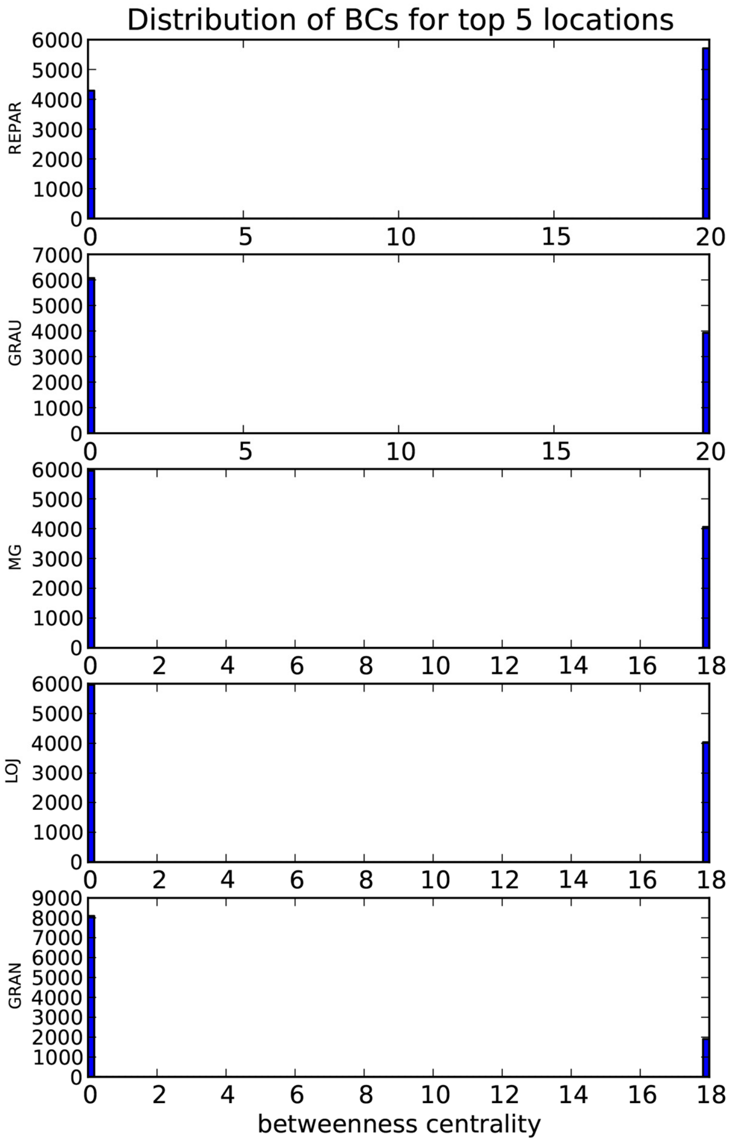

Figure S3. Distribution of betweenness centrality values for populations generated by bootstrapping using EDENetworks version 2.18.
